# Characterization of novel CD8^+^ regulatory T cells and their modulatory effects in murine model of inflammatory bowel disease

**DOI:** 10.1007/s00018-024-05378-x

**Published:** 2024-08-01

**Authors:** Jia-Ning Fan, Hsin Ho, Bor-Luen Chiang

**Affiliations:** 1 Graduate Institute of Clinical Medicine, College of Medicine, National Taiwan University, Taipei, Taiwan; 2 Department of Pediatrics, National Taiwan University Hospital, No. 7 Chung-Shan South Road, Taipei, 100 Taiwan; 3 Genome and Systems Biology Degree Program, College of Life Science, National Taiwan University, Taipei, Taiwan

**Keywords:** Treg-of-B cells, CD8^+^ tregs, Inflammatory bowel disease, DSS-induced colitis

## Abstract

Dysregulation of mucosal immune system has been proposed to be critical in the pathogenesis of inflammatory bowel diseases (IBDs). Regulatory T cells (Tregs) play an important role in regulating immune responses. Tregs are involved in maintaining intestinal homeostasis and exerting suppressive function in colitis. Our previous studies showed that a novel forkhead box protein P3 (Foxp3) negative Tregs (Treg-of-B cells), induced by culturing naïve CD4^+^ T cells with B cells, could protect against colitis and downregulate T helper (Th) 1 and Th17 cell cytokines in T cell-mediated colitis. In the present study, we aimed to induce Treg-of-B cells in the CD8^+^ T-cell population and investigate their characteristics and immunomodulatory functions. Our results showed that CD8^+^ Treg-of-B cells expressed Treg-associated markers, including lymphocyte-activation gene-3 (LAG3), inducible co-stimulator (ICOS), programmed death-1 (PD-1), cytotoxic T-lymphocyte-associated protein-4 (CTLA-4), tumor necrosis factor receptor superfamily member-4 (TNFRSF4, OX40), and tumor necrosis factor receptor superfamily member-18 (TNFRSF18, GITR), but did not express Foxp3. CD8^+^ Treg-of-B cells produced higher concentration of inhibitory cytokine interleukin (IL)-10, and expressed higher levels of cytotoxic factor granzyme B and perforin after stimulation, compared to those of CD8^+^CD25^-^ T cells. Moreover, CD8^+^ Treg-of-B cells suppressed T cell proliferation in vitro and alleviated colonic inflammation in chronic dextran sulfate sodium (DSS)-induced colitis. In conclusion, our study identified a novel subpopulation of CD8^+^ Tregs with suppressive effects through cell contact. These CD8^+^ Treg-of-B cells might have therapeutic potential for IBDs.

**Supplementary Information:**

The online version contains supplementary material available at 10.1007/s00018-024-05378-x.

## Introduction

Regulatory T cells (Tregs) play an essential role in maintaining immune tolerance and modulating immune responses. In our previous study, we found that naïve B cells from the spleen and Peyer’s patches could induce CD4^+^CD25^-^ T cells to convert into CD25^+^Foxp3^-^ T cells, which exerted suppressive effects; named Treg-of-B cells [1, 2]. Treg-of-B cells had been applied to the treatment of inflammatory bowel disease (IBD), collagen-induced arthritis, and allergic asthma [1, 3, 4]. Our studies showed that Treg-of-B cells expressed Treg-related molecules, including ICOS, LAG3, PD-1, CTLA-4, OX40, and GITR [1, 3–5]. To date, the most reliable and well-known Treg cell population is the CD4^+^Foxp3^+^ Treg population. Some subsets of CD8^+^ T cells also exhibit regulatory functions in the immune response. Based on these findings, the function and development of Tregs in the CD8^+^ T-cell population should be further investigated.

CD8^+^ Tregs are a heterogeneous population with different phenotypic characteristics and suppressive functions. The most common and well-recognized CD8^+^ Treg subsets include CD8^+^Foxp3^+^ [6–9], CD8^+^CD25^+^ [10, 11], CD8^+^CD122^+^ [12], and CD8^+^CD28^-^ T cells [13]. Different mechanisms of CD8^+^ Treg-mediated suppression had been described. CD8^+^ Tregs could release inhibitory cytokines, such as interleukin (IL)-10, IL-34, IL-35, transforming growth factor (TGF)-β, and interferon (IFN)-γ [14–18]. CD8^+^CD28^-^ T cells could block T cell activation by downregulation of co-stimulatory molecules CD86 and CD80 on antigen-presenting cells (APCs) [19, 20]. Certain subsets of CD8^+^ Tregs have a suppressive ability through cell-cell contact or cytotoxic mechanisms [6, 21–24]. In addition, CD8^+^ Tregs could also express ectoenzymes ectonucleoside triphosphate diphosphohydrolase-1 (NTPDase1, CD39) and 5ʹ-nucleotidase (5ʹ-NT, CD73) to dephosphorylate adenosine triphosphate or adenosine diphosphate, and degrade adenosine monophosphate to adenosine, which inhibit T cell activation [25].

Inflammatory bowel diseases are autoimmune diseases that include Crohn’s disease and ulcerative colitis (UC). The pathophysiological factors involved in IBD included microbiota, environmental factors, genetics, and immune dysregulation [26, 27]. Mucosal immune system dysfunction plays a critical role in IBD pathogenesis. Tregs are key modulators of peripheral tolerance and participate in the suppression of colitis. In fact, CD4^+^CD25^+^ Tregs effectively prevent colonic inflammation in CD4^+^CD45RB^hi^ T-cell-induced colitis [28]. Moreover, transplantation of a Treg-enriched population (CD4^+^CD45RB^lo^ T cells) can sufficiently attenuate the development of colitis [29]. Available evidence suggests that CD8^+^ Tregs are involved in maintaining mucosal homeostasis and exerting therapeutic effects in IBD. Lamina propria CD8^+^ T cells exhibit regulatory functions in healthy individuals, but not in patients with IBD [30]. Moreover, the number of CD8^+^ Tregs in peripheral blood is substantially decreased in the acute and remittent stages of UC [31].

In this study, we generated and characterized CD8^+^ Treg-of-B cells by culturing CD8^+^ T cells with naive B cells in the presence of anti-CD3 and anti-CD28 monoclonal antibodies (mAbs). We also investigated the characteristics and suppressive abilities of the CD8^+^ Treg-of-B cells. Furthermore, we established a chronic DSS-induced colitis model and found that CD8^+^ Treg-of-B cells exhibited immunomodulatory functions in IBD.

## Materials and methods

Materials and method**s** are provided in the supplementary information.

## Results

### The phenotypical characterization of CD8^+^ Treg-of-B cells

**Fig. 1:**
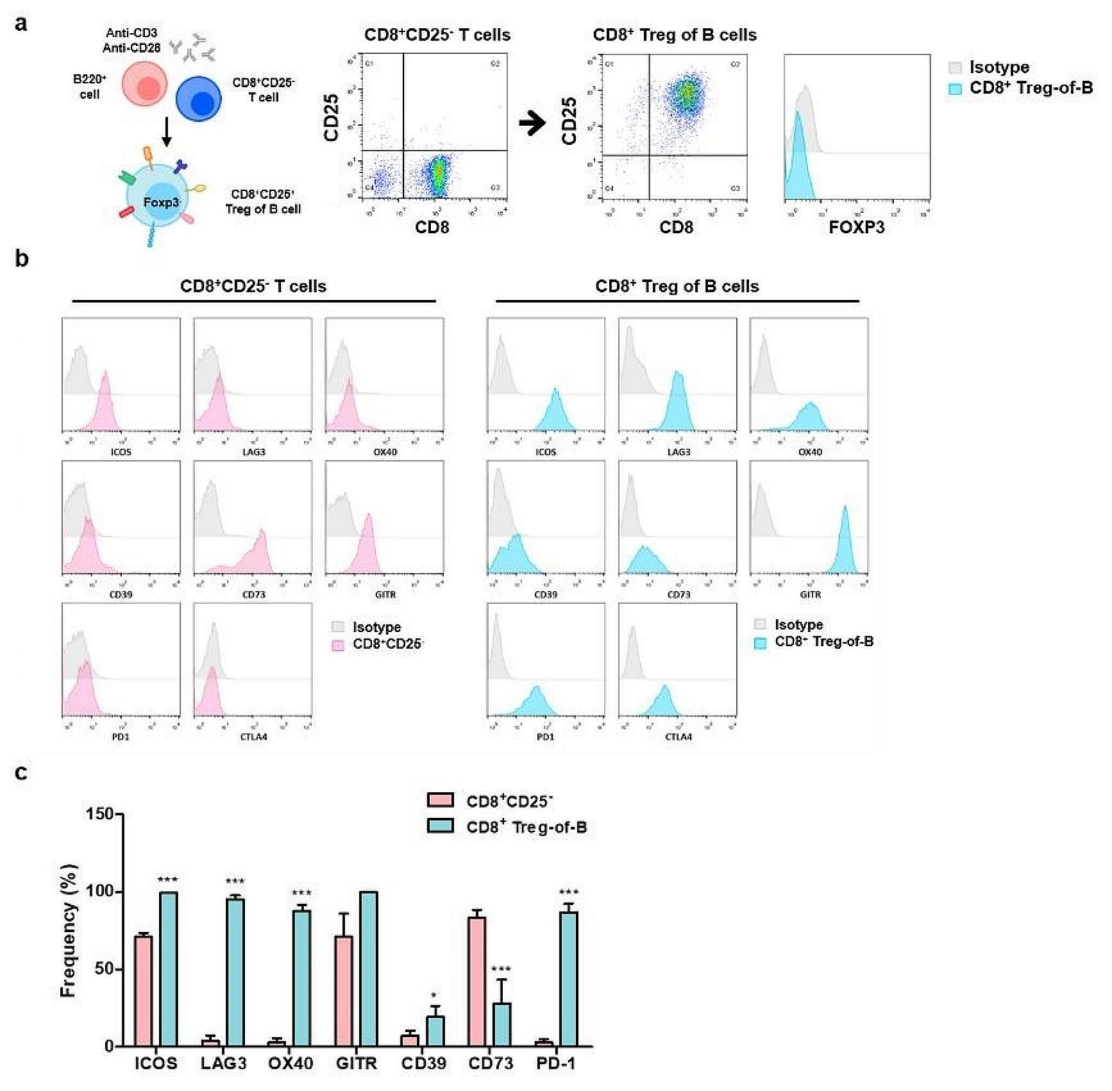
Phenotypical characterization of CD8^+^ Treg-of-B cells. **a** After co-cultured with B cells, CD8^+^CD25^−^ T cells converted into CD25^+^Foxp3^−^ Tregs and we named CD8^+^ Treg-of-B cells. **b** FACS analysis of CD8^+^CD25^−^ T and CD8^+^ Treg-of-B cells with Tregs-associated markers. **c** The data represented the mean percentage of surface marker expression by CD8^+^ Treg-of-B and CD8^+^CD25^−^ T cells. Data was presented as the mean ± SD. *, *p* ≤ 0.05; ***, *p* ≤ 0.001, compared with CD8^+^CD25^−^ T cell group. The data are representative of three to four independent experiments

To determine whether B cells had the ability to convert CD8^+^ T cells into regulatory T cells. First, we isolated CD8^+^ T and B220^+^ B cells from C57BL/6 mouse splenocytes using magnetic bead-conjugated antibodies. CD8^+^ T cells and B cells were treated with 1 µg/ml anti-CD3ε/CD28 mAbs, which provided the co-stimulatory signal. After three days cultured, CD8^+^ T cells could convert into CD8^+^CD25^+^Foxp3^−^ T cells, called “CD8^+^ Treg-of-B cells” (Fig. 1a). To characterize the CD8^+^ Treg-of-B cells, we examined the expression of Treg-related molecules by Flow cytometry analysis. Compared to naïve CD8^+^CD25^−^ T cells, CD8^+^ Treg-of-B cells expressed higher levels of Treg-associated markers, including OX40, LAG3, ICOS, CD39, PD1, and CTLA4, but lower levels of CD73 (Fig. 1b, c).

**Table 1:** The sequences of primers used for q-PCR

Gene name	Forward	Reverse
Gapdh	GATGGGTGTGAACCACGAGA	AGATCCACGACGGACACAT
Granzyme B	ACTTTCGATCAAGGATCAGCA	ACTGTCAGCTCAACCTCTTGT
Perforin	GAGAAGACCTATCAGGACCA	AGCCTGTGGTAAGCATG
IL-34	ACTCAGAGTGGCCAACATCACAAG	ATTGAGACTCACCAA GACCCACAG
IL-12p35	CGTCTTTGATGATGACCCTGTGCC	TGTAAGGGTCTGCTTCTCCCACAG

### Cytokine production and cytotoxic factor expression in CD8^+^ Treg-of-B cells

**Fig. 2:**
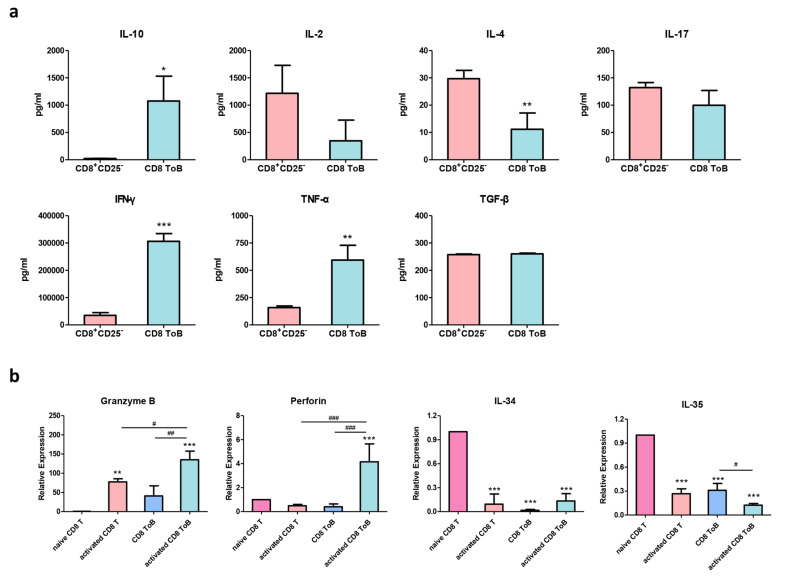
The expression of cytokines and cytotoxic molecules in CD8^+^ Treg-of-B cells. **a** The cytokine levels of CD8^+^CD25^−^ T and CD8^+^ Treg-of-B cells (CD8 ToB) in 48 h conditioned media was measured by ELISA. **b** CD8^+^CD25^−^ T and CD8^+^ Treg-of-B cells were cultured with anti-CD3/CD28 mAbs for 16 h, and the gene level of cytotoxic proteins and inhibitory cytokines was detected by qPCR. Data was presented as the mean ± SD. *, *p* ≤ 0.05; **, *p* ≤ 0.01; ***, *p* ≤ 0.001, compared with CD8^+^CD25^−^ T or naïve CD8 T cell group. #, *p* ≤ 0.05; ##, *p* ≤ 0.01; ###, *p* ≤ 0.001, ns, not significant, for select comparisons. The data are representative of three independent experiments

To examine the cytokine profiles in CD8^+^ Treg-of-B cells, CD8^+^CD25^−^ T and CD8^+^ Treg-of-B cells were cultured with anti-CD3ε/CD28 mAbs for 48 h. The conditioned media were collected, and cytokines were detected by ELISA. Higher levels of IFN-γ, IL-10, and TNF-α were secreted by activated-CD8^+^ Treg-of-B cells than that of CD8^+^CD25^−^ T cells in 48 h conditioned media (Fig. 2a). Next, we analyzed the gene expression of inhibitory cytokines and cytotoxic factors in naïve and stimulated CD8^+^ Treg-of-B cells. The primer sequences of qPCR were listed in Table 1. The results showed that granzyme B and perforin gene expression was higher in CD8^+^ Treg-of-B cells after stimulation. Compared to naïve CD8^+^ T cells, CD8^+^ Treg-of-B cells expressed lower expression of *IL-34* and *IL-35* mRNA (Fig. 2b).

### CD8^+^ Treg-of-B cells exerted their suppressive effect via direct cell contact

**Fig. 3:**
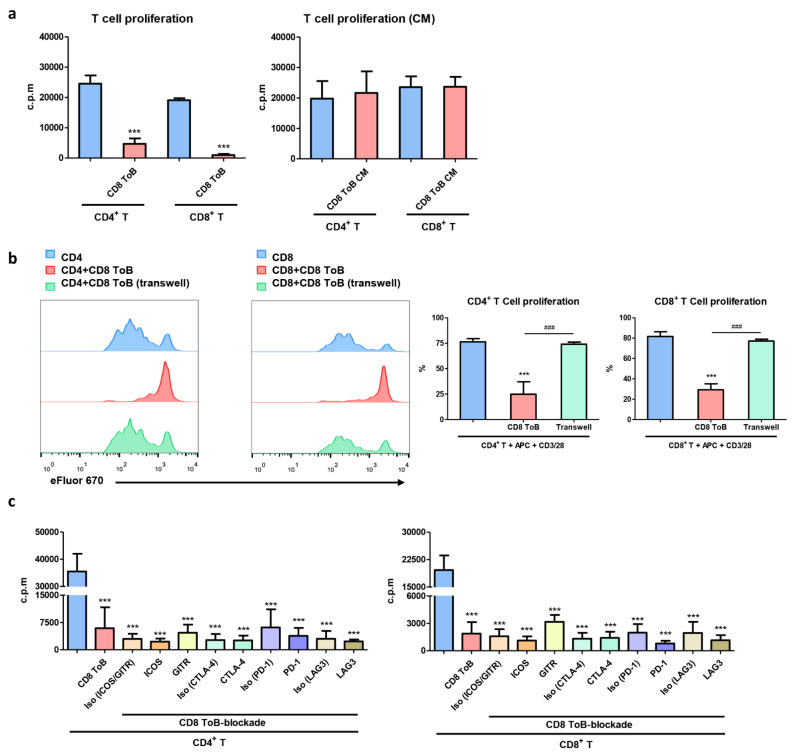
The suppressive capacity of CD8^+^ Treg-of-B cells. **a** The suppressive ability of CD8^+^ Treg-of-B cells (CD8 ToB) and conditioned media (CM) were evaluated by CD4^+^ and CD8^+^ responder T cells. The T cell proliferation was performed by 3 H-incorporation assay. **b** CD8^+^ Treg-of-B cells and eF670-labeled responder T cells were separated by using Transwell system for 72 h. Responder T cells were collected and analyzed by FACSCalibur. **c** CD8^+^ Treg-of-B cells were pre-treated by neutralizing Abs. The cell proliferation was performed by 3 H-incorporation assay. Data was represented as the mean ± SD. ***, *p* ≤ 0.001, compared with responder T cell group. #, *p* ≤ 0.05; ##, *p* ≤ 0.01; ###, *p* ≤ 0.001, for select comparisons. The data are representative of three to four independent experiments

.To examine the inhibitory effect of CD8^+^ Treg-of-B cells, responder T cells were co-cultured with mitomycin C (MMC)-treated APCs. CD8^+^ Treg-of-B cells efficiently suppress the proliferative response of both CD4^+^ and CD8^+^ T cells. In contrast, conditioned medium from CD8^+^ Treg-of-B cell did not exert a suppressive effect (Fig. 3a). Next, we used the Transwell system to study the relationship between cell contact and suppressive function, and the results showed that CD8^+^ Treg-of-B cell-mediated T cell suppression was abolished in the Transwell culture (Fig. 3b). To investigate the role of surface molecules in CD8^+^ Treg-of-B cell-mediated suppression, we used neutralizing antibodies to block the surface molecules. The results showed that blocking most of these surface molecules did not abolish the suppressive effects of CD8^+^ Treg-of-B cell in CD4^+^ and CD8^+^ T cells proliferation. Only the blockade of GITR slightly reversed CD8^+^ T cell proliferation (Fig. 3c). These results demonstrated that CD8^+^ Treg-of-B cells exerted their suppressive effect via direct cell-contact mechanisms; however, the key molecules remain unknown.

### Adoptive transfer of CD8^+^ Treg-of-B cells alleviated colitis

**Fig. 4:**
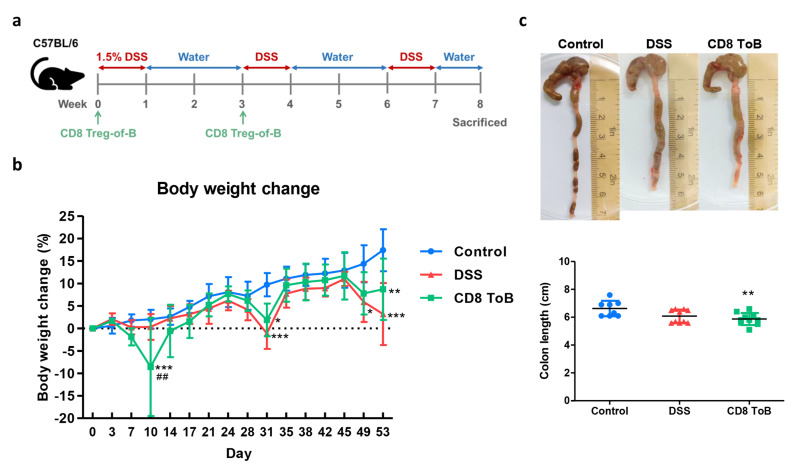
Induction of DSS-induced colitis and transplantation of CD8^+^ Treg-of-B cells. **a** Chronic DSS colitis was induced by 1.5% DSS for one week, replace DSS by drinking water for two weeks, and repeat two rounds. Mice were injected intraperitoneally with CD8^+^ Treg-of-B cells (CD8 ToB) at week 0 and 3. **b** Body weights were measured twice a week. The body weight changes were calculated. Formula: (Weight on each day - Initial weight)/Initial weight × 100. **c** On week 8, the mice were sacrificed, and colon lengths were measured. Data was represented as the mean ± SD. *, *p* ≤ 0.05; **, *p* ≤ 0.01; ***, *p* ≤ 0.001, compared with control group. ##, *p* ≤ 0.01, compared with DSS group. The sample numbers were eight to ten mice per group (*n* = 8/Control group, *n* = 10/DSS group, *n* = 10/CD8^+^ Treg-of-B cell group)

To examine the immunomodulatory function of CD8^+^ Treg-of-B cells in IBD. In this study, we established a murine model of DSS-induced chronic colitis. Mice were fed three rounds of 1.5% DSS in drinking water and injected intraperitoneally with CD8^+^ Treg-of-B cells before the first and second rounds of DSS administration (week 0 and 3; Fig. 4a). Mice that received DSS lost weight during the second and third rounds of DSS and had shorter colon lengths than control mice. Compared with DSS mice, mice adoptively transferred with CD8^+^ Treg-of-B cells showed a slight reversal in body weight loss on the day of sacrifice (Fig. 4b), but colon length did not differ significantly (Fig. 4c).

**Fig. 5:**
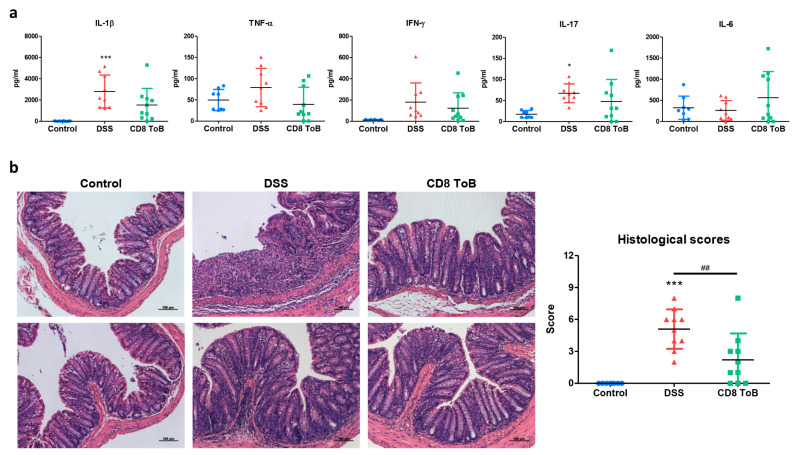
Transplantation of CD8^+^ Treg-of-B cells alleviated colitis. **a** Detected the cytokine level in 72 h cultured supernatant from colon tissue. **b** Representative images of the colon sections (H&E staining, Scale bars, 100 μm.) and histological colitis scores are shown. Data was represented as the mean ± SD. *, *p* ≤ 0.05; ***, *p* ≤ 0.001, compared with control group. ##, *p* ≤ 0.01, for select comparisons. CD8 ToB, CD8^+^ Treg-of-B cells. The sample numbers were eight to ten mice per group (*n* = 8/Control group, *n* = 10/DSS group, *n* = 10/CD8 + Treg-of-B cell group)

We further measured the production of pro-inflammatory cytokines, TNF-α, IFN-γ, IL-6, IL-1β, and IL-17 in colonic tissue of colitis mice. We collected the culture supernatant from the colonic tissue and analyzed it using ELISA. The mean levels of TNF-α and IL-1β from colon of mice adoptively transferred with CD8^+^ Treg-of-B were lower compared to DSS mice but without statistical significance (Fig. 5a). The histomorphology of colon tissue from DSS mice showed marked cell infiltration and focal ulceration. In contrast, mice that received CD8^+^ Treg-of-B cells exhibited milder histopathology in colon sections. Histological scores were evaluated based on inflammatory cell infiltration, mucosal architecture, and epithelial cell hyperplasia. The results demonstrated that adoptive transfer of CD8^+^ Treg-of-B cells alleviated colitis (Fig. 5b).

## Discussion

B cells play an essential role in the adaptive immune response and exert their function by producing immunoglobulin and secreting cytokines. Many studies also revealed the important role of B cells in the induction of T-cell tolerance [32, 33]. In addition, naive B cells could generate regulatory T cells in the presence of a mature immunologic synapse [34]. B220 is commonly used as B cell marker in mouse, and B220^+^ cells could serve as APCs in T-cell response. Our previous studies also suggested that CD4^+^ Treg-of-B cells induced by B220^+^ B cells in the presence of anti-CD3ε/CD28 mAbs [4].

Accumulating evidence on Tregs showed that the role of Foxp3^-^ Tregs, such as type 1 Tregs (Tr1) and Th3 cells, which secreted IL-10 and TGF-β respectively [35, 36]. CD4^+^ Treg-of-B cells do not express the common Treg transcription factors, Foxp3 and Helios [37]. Furthermore, CD4^+^ Treg-of-B cells are different from the recognized Foxp3^-^ Tregs. Although CD4^+^ Treg-of-B cells secreted higher amounts of TGF-β and IL-10 compared to those of naïve CD4^+^CD25^-^ T cells, TGF-β and IL-10 did not play the crucial role in Treg-of-B cell-mediated suppression [1, 2, 38]. CD8^+^ T cells co-cultured with B220^+^ cells also induced CD8^+^ Treg-of-B cells, which express phenotypic markers similar to those of CD4^+^ Treg-of-B cells (Fig. 1) and exerted the ability to inhibit T cell proliferation (Fig. 3a).

The suppressive ability of CD8^+^ T-cells was first described in 1970s by Gershon and Kondo [39, 40]. However, subsequent studies failed to differentiate *CD8*^*+*^*suppressor T cells* from other CD8^+^ T cells due to lacking the specific molecules or transcription factors. When immunosuppressive CD4^+^CD25^+^ T cells were identified [41], interest on CD8^+^ suppressor T cells was revived, and now were commonly referred to CD8^+ “^regulatory” T cells. CD8^+^ Tregs can be classified as natural and adaptive/induced [42]. Thymus-derived nature CD8^+^ Tregs had been reported to express the markers, CD25^+^GITR^+^CTLA-4^+^Foxp3^+^, and exhibit suppressive function by CTLA-4 and TGF-β-dependent mechanism [43]. In mice, natural CD8^+^ Tregs are characterized as CD8^+^CD122^+^ or Qa-1 restricted CD8αα^+^ populations [44, 45].

Several studies have shown that CD8^+^ Tregs are induced by a variety of cytokines and antigenic stimuli. Culturing CD8^+^CD28^-^ T cells with IL-2 and IL-10 can induce CD8^+^CD28^-^Foxp3^-^ Tregs, which inhibit antigen-presenting activity of dendritic cells (DCs), and cytotoxic function of cytotoxic T cell (CTL) through IL-10 [46, 47]. CD8^+^CD25^+^Foxp3^+^ Tregs can also be generated by culturing CD8^+^CD25^-^ T cells in the presence of staphylococcal enterotoxin B and anti-CD3/CD28 mAbs, which inhibit T cell proliferation via cell-cell contact mechanisms [48]. Some studies also revealed that activated DCs can induce CD8^+^ suppressor T cell generation, lipopolysaccharide-activated DC-induced CD8^+^TCRαβ^+^CD25^+^ T cells inhibited effector T cells by cell-cell interaction, and allogeneic CD40 ligand-activated plasmacytoid DCs can promote differentiation of CD8^+^ suppressor T cells, which exert suppressive function by secreting IL-10 [49]. In this study, we reported that CD8^+^CD25^-^ T cells cocultured with B cells in the presence of anti-CD3/CD28 mAbs could induce CD8^+^ Treg-of-B cells, a novel CD8^+^ Treg population which was characterized as CD8^+^CD25^+^CTLA-4^+^Foxp3^-^, and secreted high levels of IL-10, TNF-α, and IFN-γ (Figs. 1 and 2). Similar to other studies [48, 50], CD8^+^ Treg-of-B cells inhibited CD4^+^ and CD8^+^ T cell proliferation in a cell-cell contact-dependent manner, as shown in the Transwell experiments (Fig. 3b). However, the suppressive mechanisms of CD8^+^ Treg-of-B cell are still unknown. Previous studies showed that glycogen synthase kinase 3 β (GSK3β) became quickly deactivated (phosphorylated at serine 9, Ser9) during induced regulatory T cell (iTreg) differentiation [51]. In addition, inhibition of GSK3 activity promotes the generation and enhanced suppressive function of human IL-10^+^ Foxp3^+^ iTregs [52]. Hence, we examined the protein expression of total GSK3β and p-GSK3β (Ser9). However, the phosphorylation ratio of GSK3β (Ser9) was decreased in CD8^+^ Treg-of-B cells (data not shown). These results indicated that GSK3β activity was not reduced after CD8^+^ Treg-of-B cell induction.

In our previous study, we found that CD4^+^ Treg-of-B cells can alleviate the intestinal inflammation and suppress Th1 and Th17 cytokines IFN-γ, TNF-α, and IL-17 in CD4^+^CD45RB^hi^ T cell-induced colitis through an IL-10-independent mechanism [4]. Available evidence suggests that CD8^+^ Tregs play an essential role in IBD by maintaining intestinal homeostasis and suppressing immune responses. In a murine model, naturally occurring CD8^+^CD28^-^ Tregs and in vitro activated CD8^+^CD122^+^ T cells prevented CD4^+^CD45RB^hi^ T cell-induced colitis. CD8^+^ Tregs from IL-10 knockout mice lose their ability to suppress colitis, indicating that IL-10-secreting CD8^+^ Tregs play a crucial role in the prevention of IBD [53, 54]. In addition, Qa-1-restricted CD8^+^ Tregs induced by glatiramer acetate (GA) ameliorated DSS-induced colitis. GA-induced CD8^+^ Tregs exhibit suppressive functions through perforin-mediated cytotoxicity [55]. In this study, to investigate the immunomodulatory effects of CD8^+^ Treg-of-B cells in IBD, chronic DSS-induced colitis model was established. In contrast to previous studies, [53, 54] our findings showed that mice injected with CD8^+^ Treg-of-B cells alleviated weight-loss but without statistical significance (Fig. 4b), but the mean levels of IL-1β and TNF-α in colon cultures slightly decreased and the histopathology of colon sections showed significant improvements after CD8^+^ Treg-of-B cells treatment (Fig. 5). These results suggested an immunosuppressive function of CD8^+^ Tregs in patients with IBD. These discrepancies are possibly attributable to the characteristics of CD8^+^ Tregs and the differences between the experimental models of IBDs.

**Fig. 6:**
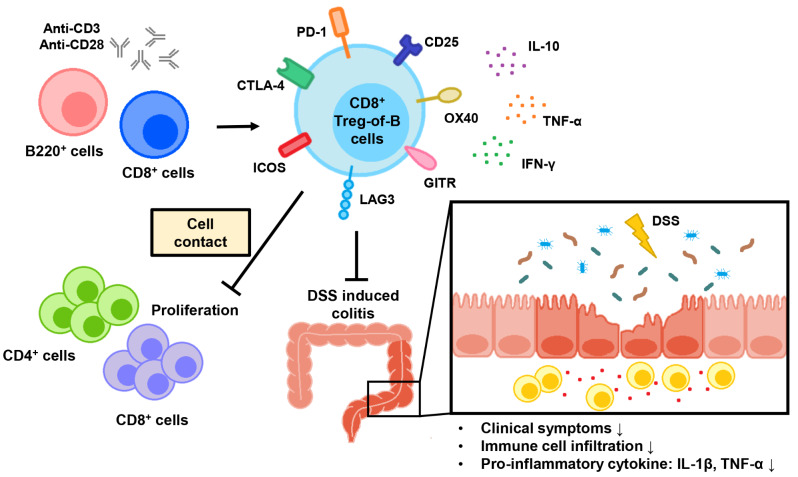
The schematic illustration described the characteristic of CD8^+^ Treg-of-B cells and their modulatory function. CD8^+^ T cells culture with B220^+^ cells in the presence of anti-CD3/CD28 mAbs could induce CD8^+^ Treg-of-B cell, which inhibited CD4^+^ and CD8^+^ T cells proliferation through direct cell contact. Transplantation of CD8^+^ Treg-of-B cell in DSS model could ameliorate the clinical severity of colitis

In conclusion, we demonstrated that CD8^+^ Treg-of-B cell is a novel CD8^+^ Treg subset with a strong suppressive function. In vitro, CD8^+^ Treg-of-B cells exerted their suppressive effects through a direct cell contact mechanism. In vivo, CD8^+^ Treg-of-B cells decreased the inflammatory cell infiltration of the mucosa in DSS-induced colitis (Fig. 6). Although the mechanisms by which CD8^+^ Treg-of-B cells alleviated colitis were unclear in this study, our findings suggested that CD8^+^ Treg-of-B cells might participate in regulating intestinal homeostasis and support the therapeutic potential of CD8^+^ Tregs in IBD. The critical molecules which involved in the development and function of CD8^+^ Treg-of-B cells are to be identified in future studies.

### Electronic supplementary material

Below is the link to the electronic supplementary material.

Supplementary Material 1

## Data Availability

The data used and analyzed during the current study are available from the corresponding author on reasonable request.
