# Supplementary material for: Characterization of novel CD8+ regulatory T cells and their modulatory effects in murine model of inflammatory bowel disease
Source: Cell Mol Life Sci. 2024 Aug 1;81(1):327. doi: 10.1007/s00018-024-05378-x (PMC11335251; doi:10.1007/s00018-024-05378-x)
Supplement: Supplementary file 1 — Supplementary Material 1 [file 18_2024_5378_MOESM1_ESM.docx]

**Journal name: Cellular and Molecular Life Sciences**

**Characterization of Novel CD8^+^ Regulatory T Cells and Their Modulatory Effects in Murine Model of Inflammatory Bowel Disease**

**Jia-Ning Fan^1^, Hsin Ho^1^, and Bor-Luen Chiang^1, 2, 3^**

^1^Graduate Institute of Clinical Medicine, College of Medicine, National Taiwan University, Taiwan

^2^Department of Pediatrics, National Taiwan University Hospital, Taipei, Taiwan.

^3^Genome and Systems Biology Degree Program, College of Life Science, National Taiwan University, Taipei, Taiwan

**Correspondence to:**

Dr. Bor-Luen Chiang

Department of Pediatrics

National Taiwan University Hospital

No. 7 Chung-Shan South Road,

Taipei, 100, Taiwan

E-mail: gicmbor@ntu.edu.tw

Tel: +886-2-2312-3456 ext. 267302

Fax: +886-2-2311-9087

**Materials and Methods**

**Mice**

The 6-8 weeks old C57BL/6 mice were purchased from the National Laboratory Animal Center, and maintained in specific pathogen-free condition. The study protocols were approved by the Institutional Animal Care and Use Committee of the College of Medicine, National Taiwan University. The mice were euthanized with carbon dioxide.

**Cell preparation**

Mice splenocytes were suspended and lysed. B cells were isolated by positive selection labeling with B220 magnetic beads (anti-mouse CD45R/B220 magnetic particles, BD PharMingen). CD4^+^ and CD8^+^ T cells were separated by using CD4^+^/CD8^+^ T cell isolation cocktail (EasySep^TM^ Mouse CD4^+^/CD8^+^ T cell isolation kit, STEMCELL^TM^) according to the manufacturer's manual. The total splenocytes were pre-treated with 25 µg/ml mitomycin C (MMC, Sigma–Aldrich) in 37℃ for 1 h as APCs.

**Generation of CD8^+^ Treg-of-B-cells**

Equal numbers of CD8^+^ T and B220^+^ cells were cultured in culture medium (RPMI 1640 supplemented with 5% Fetal bovine serum, Gibco) in presence of 1μg/ml anti-CD3ε and anti-CD28 (BioLegend) mAbs. After 3 days incubation, all cells were collected and removed dead cell by Ficoll-Paque (GE Healthcare). CD8^+^ Treg-of-B-cells were further enriched by using B220 microbeads to deplete B cells.

**Suppression assays**

The activation of responder T cells (freshly purified CD4^+^ and CD8^+^ T cells) was mediated by MMC-treated APCs and anti-CD3ε/CD28 mAbs. CD8^+^ Treg-of-B, responder T cells, and MMC-treated APCs were cultured at a ratio of 1:1:1 in 96-well round bottom plates. In antibody blocking experiments, CD8^+^ Treg-of-B cells were pre-treated with 20μg/ml anti-ICOS (BD), anti-GITR (BD), anti-CTLA-4 (BioLegend), anti-PD-1 (BD), anti-LAG3 (BD), and the relative isotype control antibodies for 1 h before seeded with responder T cells. After 3 days cultured, cells were pulsed with 3H-thymidine (PerkinElmer) and cultured for another 16-18 h. The incorporation of 3H-thymidine was detected by β-counter (PerkinElmer). The results were presented as counts per minute (c.p.m).

**Transwell assays**

Transwell assays were conducted with 1.0 μm pore size membranes (Millipore) in 24-well plates. The semipermeable membrane which separates the lower and upper chambers allows diffusion of soluble factors but not cells. Cell proliferation dye eFluor 670 (eF670, eBioscience™)-labeled responder T cells were cultured with MMC-treated APCs in the lower compartment, while CD8^+^ Treg-of-B cells were seeded in the upper compartment. After incubated 3 days, responder T cells were analyzed by FACSCalibur (BD).

**Enzyme-linked immunosorbent assay (ELISA)**

CD8^+^ Treg-of-B and CD8^+^CD25^-^ T cells (1x10^5^) were stimulated with anti-CD3ε/CD28 mAbs for 48 h. Conditioned media were collected and assessed by ELISA kit (R&D), according to the manufacturer’s instructions.

**Reverse transcription and quantitative PCR (qPCR)**

The pellets of cells were lysed by REzol^TM^ C&T (PROtech). Total RNA was purified by phenol-chloroform extraction and reverse transcribed using RevertAid Reverse Transcriptase (Thermo). qPCR was conducted by StepOnePlus^TM^ Real-Time PCR System (Applied Biosystems™) with IQ2 SYBR Green (Bio Genesis Technologies). Relative levels of RNA were calculated and data were presented as normalized to housekeeping gene *GAPDH*. The primer sequences of qPCR were listed in Table 1.

**Flow cytometry**

To characterize the cells phenotype, following fluorescence-conjugated anti-mouse mAbs were used: B220, CD8, CD4, CD25, PD-1, ICOS, LAG3, OX40, GITR, CD39, CD73, CTLA-4, Foxp3 and the respective isotype controls. For intracellular staining, cells were fixed by Fixation Buffer (BD Pharmingen) and washed with 1X Permeabilization Buffer (eBioscience). Cells were analyzed by FACSCalibur (BD).

**DSS-induced colitis and adoptive transfer of CD8^+^ Treg-of-B cells**

Eight weeks male C57BL/6 mice were used to induce chronic colitis model. Before the experiments, all mice were weighed and equilibrate average group body weights. Mice were provided with 1.5% DSS (MP Biomedicals) in drinking water for one week, and the DSS solution was replaced with drinking water for two weeks, and repeated two rounds. After establishment of DSS-induced colitis model, mice were infused with CD8^+^ Treg-of-B cells (3x10^6^) by intraperitoneal injection at week 0 and 3. Body weight was measured twice a week, and mice were sacrificed on week 8. Calculated the percentage of body weight change using the formula: (Weight on each day - Initial weight)/Initial weight × 100. On the day of sacrifice, the colon lengths were measured and colonic tissues were collected for histological evaluation and inflammatory cytokine detection.

**Colonic tissue explant cultures and cytokine detection**

Cut colon sections (2 cm) from the middle part, halved longitudinally, and removed the feces by flushing with cold PBS containing 1% Penicillin-Streptomycin-Amphotericin B Solution (Biological Industries). The colonic tissue was then incubated in 1ml complete RPMI 1640 medium (supplemented with 5% Fetal bovine serum, 1% PSA, 1% L-glutamine, 1% HEPES) at 37℃ for 3 days. The supernatant was collected after centrifuge. Proinflammatory cytokines including IFN-γ, IL-6, IL-1β, IL-17, and tumor necrosis factor (TNF)-α levels in conditioned medium were detected by ELISA kit according to the manufacturer’s protocol.

**Histological evaluation**

After washed with PBS, the colon fragment (1 cm proximal to the anus) was fixed with 10% neutral buffered formalin solution (cis-bio) and embedded with paraffin. The sections were stained with hematoxylin and eosin (H&E), and observed the colonic pathology. Histological changes were scored as follows: 1) severity of inflammation: 0, none; 1, mild, inflammatory cell infiltrates into mucosa; 2, moderate, inflammatory cell infiltrates into mucosa and submucosa; 3, marked, transmural inflammation; 2) intestinal architecture: 0, normal; 1, focal erosions; 2, focal ulceration; 3, extensive ulcerations. 3) hyperplasia: 0, none; 1, mild; 2, moderate; 3, marked.

**Statistical analysis**

The experimental results and analyses were performed by using GraphPad Prism 5 software. The data were presented as mean ± standard deviation (SD). Two-group comparisons were analyzed by using unpaired t-test. Groups of three or more were analyzed by ANOVA with Bonferroni's test. A *p* value ≤ 0.05 was considered significant between two different groups.
